# Enhanced Flexible Tubular Microelectrode with Conducting Polymer for Multi-Functional Implantable Tissue-Machine Interface

**DOI:** 10.1038/srep26910

**Published:** 2016-05-27

**Authors:** Hong-Chang Tian, Jing-Quan Liu, Xiao-Yang Kang, Long-Jun Tang, Ming-Hao Wang, Bo-Wen Ji, Bin Yang, Xiao-Lin Wang, Xiang Chen, Chun-Sheng Yang

**Affiliations:** 1 National Key Laboratory of Science and Technology on Micro/Nano Fabrication Laboratory, Shanghai Jiao Tong University, Shanghai, P.R. China; 2 Key Laboratory for Thin Film and Micro fabrication of Ministry of Education, Shanghai Jiao Tong University, Shanghai, P.R. China; 3 Collaborative Innovation Center of IFSA, Department of Micro/Nano-electronics, Shanghai Jiao Tong University, Dongchuan Road 800, Shanghai, 200240, P.R. China

## Abstract

Implantable biomedical microdevices enable the restoration of body function and improvement of health condition. As the interface between artificial machines and natural tissue, various kinds of microelectrodes with high density and tiny size were developed to undertake precise and complex medical tasks through electrical stimulation and electrophysiological recording. However, if only the electrical interaction existed between electrodes and muscle or nerve tissue without nutrition factor delivery, it would eventually lead to a significant symptom of denervation-induced skeletal muscle atrophy. In this paper, we developed a novel flexible tubular microelectrode integrated with fluidic drug delivery channel for dynamic tissue implant. First, the whole microelectrode was made of biocompatible polymers, which could avoid the drawbacks of the stiff microelectrodes that are easy to be broken and damage tissue. Moreover, the microelectrode sites were circumferentially distributed on the surface of polymer microtube in three dimensions, which would be beneficial to the spatial selectivity. Finally, the *in vivo* results confirmed that our implantable tubular microelectrodes were suitable for dynamic electrophysiological recording and simultaneous fluidic drug delivery, and the electrode performance was further enhanced by the conducting polymer modification.

Implantable biomedical microdevices enable the long-term operation in living human bodies, which can facilitate the restoration of body function and improvement of health condition^1^. As the interface between artificial machines and natural tissue, microelectrodes play significant roles in determining the overall efficacy of the whole implantable system^2^,^3^. In order to interact with living tissue effectively and friendly, the implantable microelectrodes needed to be: 1) tiny, to minimize the tissue damage and reduce the power consumption, which meant the cross section diameter of the electrode should be restrained in hundreds of micrometers; 2) effective, to operate with excellent performance, such as low impedance, high charge storage capacity and high signal-to-noise ratio during electrophysiological signal recording; and 3) biocompatible, to functionalize in body safely without inducing severe tissue reaction. With the development of microfabrication technologies, various kinds of microelectrodes with high density and tiny size were developed to undertake precise and complex medical tasks through electrical stimulation and electrophysiological recording^4^,^5^,^6^. Some clinical applications, such as relieving the symptoms of the Parkinson’s disease by deep brain stimulation^7^, restoring the moving functions of the paralyzed limb through functional electrical stimulation^8^, rebuilding the visual or auditory capability by utilization of the visual prosthesis and the cochlear implants^9^,^10^, had been successfully achieved under the assistance of microelectrodes.

Due to the well compatibility with the microfabrication process, stiff silicon or SU-8 microelectrodes, such as Michigan neural probes and Utah electrodes array, were popular in central nerve prostheses applications^11^,^12^. However, if there was only electrical interaction between the electrodes and the muscle or nerve tissue without nutrition factor delivery, it would eventually lead to the denervation-induced skeletal muscle atrophy^13^,^14^,^15^. In recent years, stiff microelectrodes integrated with micro channels for fluidic drug delivery were developed to provide multi-functional tissue-machine interface for electrophysiological research and applications^16^,^17^,^18^. However, because of the tremendous difference in mechanical property between the rigid microelectrode and soft tissue, the stiff microelectrodes would easily damage the living tissue or be broken by physical contact during the implantation process. Although many kinds of flexible or even stretchable microelectrodes had been developed for neural or muscular implant^19^,^20^,^21^,^22^, only a few researchers focused on the development of multi-functional flexible microelectrodes that integrated microfluidic channels as chemical interface, which could be made of flexible polymers, such as polyimide, polydimethylsiloxane (PDMS) and parylene^23^,^24^,^25^. These flexible microelectrodes with microfluidic channels were designed with the electrode sites that distributed on one side of the flat electrode structure, and without electrode-tissue interface materials which coated on the electrode sites. As a consequence, the effective range of stimulation current and electrochemical characteristic of the electrodes was restricted, respectively. Under this circumstance, the objective of our study was to develop a new type of microelectrode, which could cover the shortage of the existing flexible microelectrode with microfluidic channels.

In this paper, we developed a novel tubular microelectrode with excellent flexibility for dynamic tissue implant and tested it in the neuromuscular system. The advantages of the tubular microelectrode were as follow. First, the whole microelectrode was made of biocompatible polymers, comprising of flexible polyimide and parylene, which could avoid the drawbacks of the stiff microelectrodes that easy to be broken and damage tissue. Moreover, the microelectrode sites were circumferentially distributed on the surface of polymer capillary in three dimensions, which was different from previous designed electrode sites that distributed on one plane only^23^,^24^,^25^. The 3D positioned electrode sites were able to interact with the tissue which contact the tube electrode from any direction, while the planar distributed electrode sites could only interact with the side of the tissue which contact with the electrode sites. Also, the 3D distribution of electrode sites would benefit the spatial selectivity, which generally indicated the ability of the microelectrodes to precisely interact with a comparatively small volume of tissue in a specific direction^2^. Furthermore, different from our previous work^26^, multiple electrical channels were integrated on one flexible tube with less than 350 μm of its outer diameter. In addition, conducting polymer was electrochemically deposited on the electrode sites of the developed microelectrode to enhance its performance, which included the reduction of the impedance and the increase of the charge storage capacity.

## Results and Discussion

**Figure 1:**
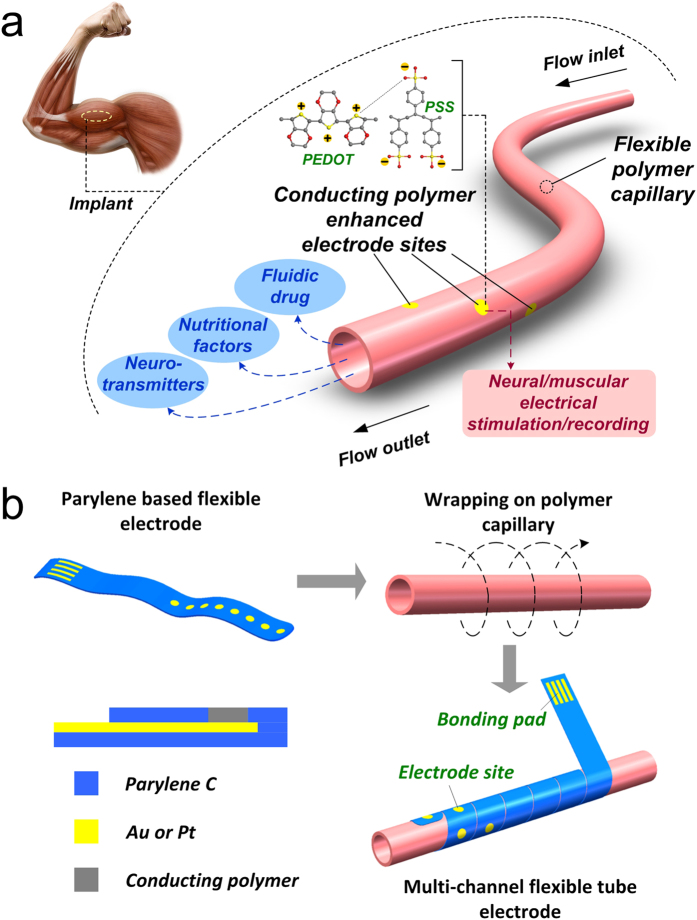
Schematic illustration of the flexible tubular microelectrode and the corresponding fabrication processes. (**a**) The construction and multi-function illustration of the flexible tubular microelectrode, comprising multi-channel electrode sites for electrical stimulation/recording and fluidic channel for drug delivery. (**b**) The fabrication process of the tubular microelectrode, mainly including: fabrication of parylene thin film microelectrode, wrapping and gluing on the polyimide capillary, and electrochemical deposition of conducting polymer.

The overall construction and function of the tubular microelectrode was illustrated in Fig. 1a. The microelectrode had hollow tubular structure in the core acting as the fluidic channel, and the electrode sites were distributed on the surface of the tubular structure which enabled the contact with tissue from any direction. With this design, the electrical stimulation sequence and electrophysiological signals could be imported and extracted through electrode sites to frame electric path, respectively. In addition, fluidic substances, such as neuro-transmitters, nutritional factors and fluidic drugs, were able to pass through the tubular structure into tissue to establish chemical modulatory mechanism. The tubular framework of microelectrode was mainly composed of two biocompatible flexible polymers: polyimide for capillary and parylene for thin film electrode. The composition and characteristics of these materials facilitated the tubular microelectrode to comply with the dynamic muscle and nerve tissue and implant safely in living tissue for a period of time^19^. Besides, poly (3,4-ethylenedioxythiophene)/ poly (sodium 4-styrenesulfonate) (PEDOT/PSS) as conducting polymer was electrochemically deposited on the electrode sites to improve the electrochemical performance, including the electrochemical impedance and the charge storage capacity, of the microelectrode due to its excellent comprehensive property (electrochemical property, stability and biocompatibility) which was evaluated in our previous work^27^.

As depicted in Fig. 1b, the fabrication process was mainly composed of: 1) fabrication of the sandwich-structured thin film electrode with multiple electrode channels based on parylene and, 2) wrapping the parylene thin film electrode on the surface of polyimide capillary and gluing them together by biocompatible tissue adhesive. The detailed fabrication process of the parylene thin film electrode was described in our previous work and illustrated in Fig. S1^28^. In this work, the polyimide capillary was with 310 μm of its outer diameter dimension and 25 μm of wall thickness. The thickness of parylene thin film electrode was approximately 10 μm, and the diameter of each electrode site of the four-channel microelectrode was 200 μm. All these dimension parameters in this work were adjusted to the following intramuscular implantation and physiological experiment, which could be flexibly varied depending on specific application.

**Figure 2:**
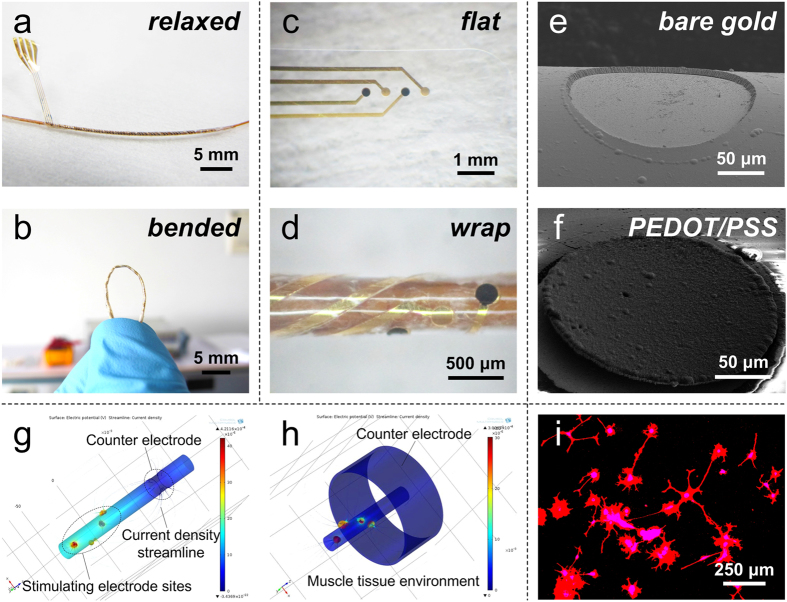
(**a,b**) Prototype of the overall relaxed and bended tubular microelectrode, respectively. (**c,d**) The electrode sites of the thin film microelectrode and the tubular microelectrode, respectively. The golden and black sites were bare gold and PEDOT/PSS modified electrode sites, respectively. (**e,f**) Enlarged view of single electrode site morphology of bare gold and PEDOT/PSS modified electrode sites, respectively. (**g,h**) finite element simulation results of current streamline distribution for small and large reference electrode configuration, respectively. (**i**) Immunofluorescent image of PC-12 cells cultured for 7 days with the addition of neural growth factor (NGF).

The prototype of fabricated tubular microelectrode was shown in Fig. 2a, and it could be bended into a circle as shown in Fig. 2b, which exhibited the excellent flexibility of the tubular microelectrode. As seen from Fig. 2c, four electrode sites were interconnected with four-channel parylene thin film electrode separately and arranged in a line, which could be subsequently wrapped on the surface of polyimide capillary to form the spatially staggered distribution (Fig. 2d). The distribution pattern of electrode sites could be arbitrarily regulated by changing their design and the wrapping way. In order to compare the electrochemical performance with the bare gold electrode sites, selective electrode sites were electrochemically deposited with PEDOT/PSS. The morphology of single electrode site without (Fig. 2e) and with (Fig. 2f) conducting polymer modification was observed by scanning electron microscopy (SEM). The thickness of the PEDOT/PSS coating was approximate 2.4 μm for thick film (shown in Fig. 2f) and 1.1 μm for thin film. It could be clearly observed that the electrode sites complied with the curved surface of polyimide capillary well, which could facilitate the contact of electrode sites with tissue. Furthermore, the conducting polymer coated on the electrode sites formed uniform morphology without fragmentation and exfoliation during the wrapping procedure. During the repeated wrapping procedure, the successfully wrapped electrode without any fragmentation or exfoliation was more than 90% among all samples. In this study, the width of the belt-like flat microelectrode was set as 2 mm to facilitate the wrapping procedure. In terms of this setup, when the included angle between the capillary and the flat microelectrode was larger than 27.6°, part of the electrode sites deposited with conducting polymer would be covered. Even so, the arbitrary spatial patterning of electrode sites could be achieved by changing the width or pattern of the belt-like flat microelectrode. In addition, as the radian of the electrode sites deposited with conducting polymer depended on the diameter of the capillary, the capillary with diameter less than 260 μm would induce the failure of the deposited polymers. Nevertheless, the failure problem could be solved by reducing the dimension of the electrode sites.

In order to investigate the diffusion range and effective region of stimulation current, the distribution of current streamline was simulated by utilizing the finite element analysis software (COMSOL Multiphysics). The simulation model was established exactly according to the arrangement of the actual fabricated tubular microelectrode that four electrode sites were helically distributed on the surface of the capillary. Moreover, two different kinds of reference electrode arrangements were involved in the simulation. One situation was that the reference electrode was arranged on the tubular microelectrode with the same outer diameter (Fig. 2g). The other situation was that the reference electrode was arranged out of the muscle bundle, whose outer diameter was larger than that of the tubular microelectrode (Fig. 2h). As demonstrated from the simulation results (Fig. S2), for both reference electrode configurations, the stimulation current was confined in the region between electrode sites and reference electrode. Furthermore, since the strongest stimulation current was restrained in the range of electrode sites, the spatial selectivity of stimulation mainly depended on the dimension and arrangement of the electrode sites. As the setup described by Metz *et al.*^23^, the stimulation spatial selectivity was about 250 μm^2^ and in one direction; for the tubular microelectrode presented here, the stimulation spatial selectivity was in four directions, though the value was larger (about 3140 μm^2^). In addition, in order to compare the different effect of electrode-tissue interaction between the situation that the electrode sites circumferentially distributed in three dimensions and that the electrode sites flatly distributed on one plane only, the flat microelectrode model was also simulated. For the tubular microelectrode (Fig. 2g), the tissue contact with the electrode from any direction could be stimulated. However, in term of flat electrode (Fig. S2g–j), the microelectrode could only interact with the tissue that situated on the side with electrode sites.

Based on the testing results in our previous study, we additionally cultured rat pheochromocytoma PC-12 cells (PC-12 cells) on the PEDOT/PSS deposited parylene thin film electrodes for 7 days^27^. As seen from Figs 2i and S3, the PC-12 cells became neural cells shape and grew closely to each other to form neurite-like interconnections with the addition of neural growth factor (NGF). The morphology of well-grown PC-12 cells indicated the good biocompatibility of developed microelectrodes.

The conducting polymer (PEDOT/PSS) was electrochemically deposited on the electrode surface to improve the electrical performance while conducting electrical stimulation and electrophysiological recording. Briefly, the deposition process started with the oxidation of EDOT (3,4-ethylenedioxythiophene) monomer to polymer chain. Then, the positively charged PEDOT was combined with the negatively charged PSS by ion bonds. Finally, the accumulated PEDOT/PSS was deposited on the electrode sites to form modification coating (Fig. 2f). The electrochemical performances of electrochemical impedance spectrum (EIS) and cyclic voltammogram (CV) were significant factors to evaluate an implantable microelectrode.

**Figure 3:**
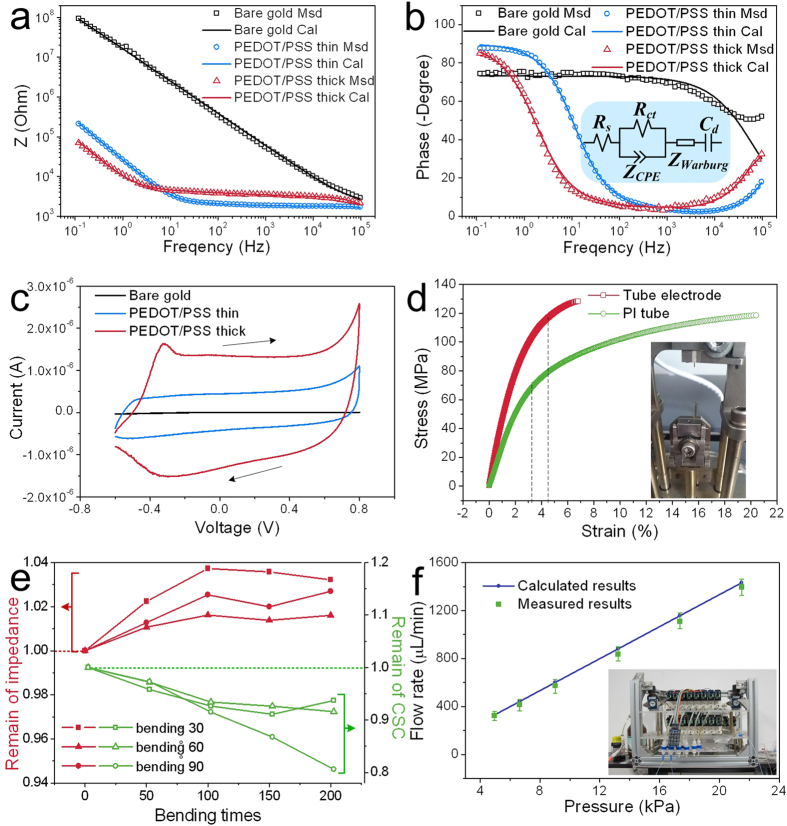
(**a,b**) Impedance plot and phase plot of electrochemical impedance spectra (EIS) of the tubular microelectrodes with and without PEDOT/PSS modification, respectively. Msd and Cal in the figure legend referred to the measured result and fitting result by equivalent circuit, respectively. The insertion indicated the equivalent circuit for EIS fitting. (**c**) Cyclic voltammograms (CV) of the tubular microelectrodes with and without PEDOT/PSS modification. The arrows indicated the cyclic scanning direction. (**d**) Strain-stress test results of the tubular microelectrode (red) and polyimide capillary (green). The insertion referred to the broken moment on the test equipment. (**e**) Remain of impedance (red) and CSC (green) of the tubular microelectrodes after repeatedly bending to the angle of 30°, 60° and 90° for 50, 100, 150 and 200 times, separately. (**f**) Flow resistance tested (green) and calculated (blue) results. The insertion referred to the multi-channel fluidic test equipment.

Firstly, as shown in Fig. 3a, the impedance of electrodes modified with PEDOT/PSS decreased in the entire frequency range compared with the bare gold electrodes. It was noticed that the impedance at 1 kHz was related to the frequency of neuronal recording and power consumption during electrical stimulation process^29^. The impedance at 1 kHz sharply decreased from 55349 ± 1043 Ω (n = 4) for bare gold electrodes to 1863 ± 244 Ω (n = 4) for PEDOT/PSS thin film coated electrodes and 3508 ± 313 Ω (n = 4) for PEDOT/PSS thick film coated electrodes. The phase angles of bare gold electrode were barely changed in the whole frequency range and mainly distributed in the range of 40°~80° (Fig. 3b). The phase angles of PEDOT/PSS modified electrodes were approximated 0° at high frequencies and 90° at low frequencies, which indicated that the PEDOT/PSS coating acted as resistive material and capacitive material, respectively. As illustrated in the insertion of Fig. 3b, the equivalent circuit was used to fit the measured impedance spectrum of electrodes with or without PEDOT/PSS modification for further analysis. As presented in Table 1 calculated from the fitting results, it was noticed that the value *n* of constant phase element (*Z* (CPE)) decreased with the increment of PEDOT/PSS coating. Since the constant phase element became pure resistance and pure capacity when the value *n* equaled to 0 and 1, respectively, the decreased *n* indicated the electrode-tissue interface formed on the electrode sites gradually became resistive material from capacitive material. This result was in agreement with the characteristics obtained from the measured impedance phase plot. Moreover, the value *B* of *Z*_Warburg_ was directly related to the diffusion time constant *τ*_*D*_, which increased with the increment of PEDOT/PSS coating. As the rose of diffusion time constant, the ion exchange rate in the electrode-tissue interface would slow down, which was the main reason that the impedance of PEDOT/PSS thick film coated electrodes in high frequency range was larger than that of PEDOT/PSS thin film coated electrodes.

Secondly, cyclic voltammograms (CV) of bare gold electrodes and PEDOT/PSS modified electrodes were measured for the investigation of redox behavior and charge storage capacity (CSC) property. As demonstrated in Fig. 3c, no obvious redox peak was observed during the CV scanning process, which indicated that the PEDOT/PSS coated electrodes possessed large pseudo capacitance. The CSC was defined as the amount of charge accumulated during the reduction scanning process, which was equal to the enveloped area of the CV curve below the baseline of zero. Quantitatively, the CSC increased from 1.25 ± 0.73 mC/cm^2^ (n = 4) for bare gold electrodes to 34.59 ± 5.62 mC/cm^2^ (n = 4) for PEDOT/PSS thin film coated electrodes and 93.92 ± 7.45 mC/cm^2^ (n = 4) for PEDOT/PSS thick film coated electrodes. The highly increased CSC mainly attributed to the excellent (inherent) electrochemical characteristic of PEDOT/PSS coating and the enlarged effective surface area of electrode sites.

Due to the extension and contraction of muscle, the tubular microelectrode would be stretched and compressed during the implantation. Therefore, we tested the mechanical property of the developed tubular microelectrode and polyimide capillary. As demonstrated in Fig. 3d, the Young’s modulus of tubular microelectrode was 4.62 GPa, which was larger than that of polyimide capillary (2.76 GPa). Furthermore, the tubular microelectrode and polyimide capillary began to yield plastic deformation when the strain increased by 4.9% and 3.7%, respectively. The improvement of mechanical performance of the developed tubular microelectrode mainly attributed to the parylene thin film electrode wrapped on the capillary surface and the tissue glue between them. Besides, in order to evaluate the mechanical stability of our developed tubular microelectrode, we repeatedly bended the tubular microelectrode modified with PEDOT/PSS to the angle of 30°, 60° and 90° for 50, 100, 150 and 200 times, separately. As shown in Fig. 3e, both the impedance and CSC of the tubular microelectrodes was barely changed when it was bended to the angle of 30° and 60° for less than 200 times. Only the CSC of the tubular microelectrodes bended to the angle of 90° for 200 times decreased about 20% compared with the initial value. This indicated the tubular microelectrode was with relatively good mechanical stability.

To study the fluidic drug delivery performance of the polyimide channel, we further tested and analyzed the flow resistance of our tubular microelectrode. As shown in Fig. 3f, the volume flow rate squeezed from the fluidic channel was approximately proportional to the applied pressure. The ratio of pressure and volume flow rate was defined as the flow resistance of the channel (*R*_f_ = Δ*p*/*Q*), which could be calculated by the inherent dimension parameters of the fluidic channel:^16^,^30^


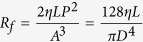


where *η* was the dynamic viscosity of the fluid, and *L*, *P* and *A* referred to the length, hydraulic perimeter and effective cross-section area, respectively. *D* represented the diameter of the circular tube. Here, the test result of flow resistance for the channel with 260 μm in diameter and 10 cm in length was (2.056 ± 0.128) × 10^13^ Pa·s/m^3^, and the calculated result was 2.214 × 10^13^ Pa·s/m^3^. The small difference between measured result and calculated result was probably due to the wall extension of tubular microelectrode. In the subsequent studies, the characteristic of controlled release of nutrients and drugs could be further enhanced by using porous and permeable polymers wrapping on the electrodes.

**Figure 4:**
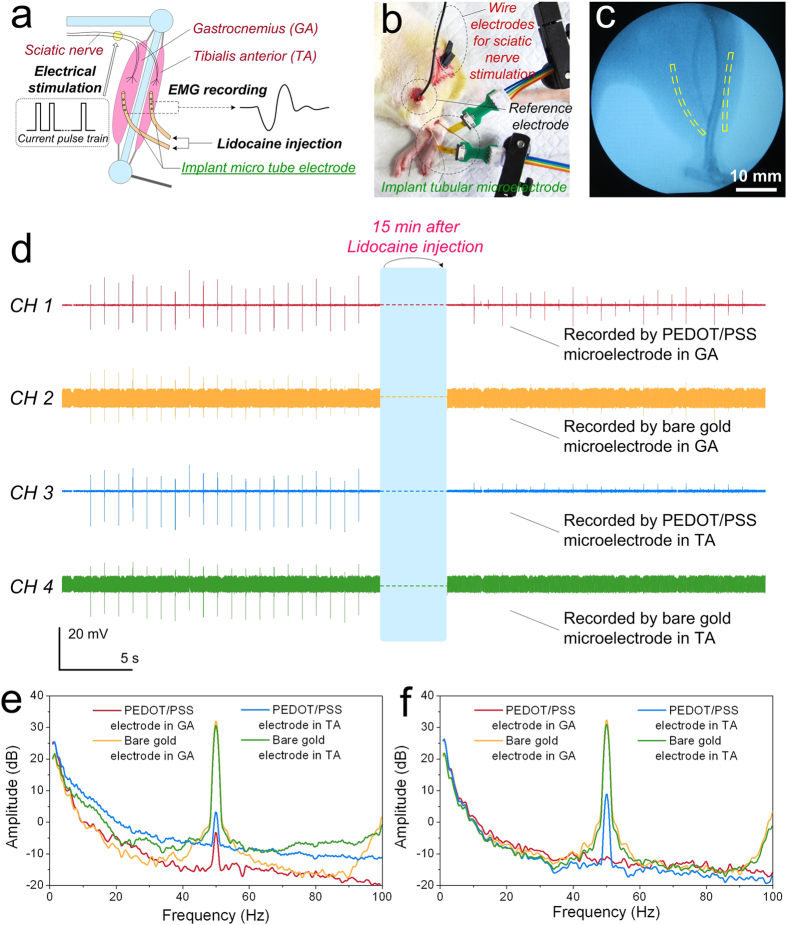
The electrophysiological experiment configuration and results *in vivo*. (**a,b**) Illustrative and actual configuration of the electrophysiological experiment, respectively. (**c**) X-ray image of the rat hind leg, in which the yellow dash line indicated the location of implanted tubular microelectrodes in gastrocnemius (GA) and tibialis anterior (TA). (**d**) EMG signals recorded by PEDOT/PSS modified electrode in GA (CH1), bare gold electrode in GA (CH2), PEDOT/PSS modified electrode in TA (CH3) and bare gold electrode in TA (CH4). The waveforms on the left and right referred to the EMG signal recording before and after lidocaine injection, respectively. (**e,f**) Frequency spectrum analysis of EMG signals before and after lidocaine injection, respectively.

To study the practical efficacy of our tubular microelectrode *in vivo*, we implanted two tubular microelectrodes into gastrocnemius (GA) and tibialis anterior (TA) of a rat, separately. As illustrated in Fig. 4a, the electrophysiological experiment mainly consisted of three parts: 1) electrical stimulation of sciatic nerve by a pair of wire electrodes made of platinum; 2) electromyography (EMG) recording by the electrical path of the tubular microelectrodes; 3) fluidic drug injection through the fluidic channel of the tubular microelectrode. The actual experimental setup on rat according to the schematic diagram was presented by Fig. 4b and Fig. S4, while the location of implanted tubular microelectrodes in GA and TA was indicated in the X-ray graph (Fig. 4c). When applying the stimulation of current pulse to the sciatic nerve of rat, the corresponding muscles from hind leg would contract and generate the EMG signal, as it is called M-wave^13^,^14^,^15^. Here, we gradually increased the amplitude of current pulse from 0.2 mA to 2 mA to figure out the current amplitude threshold which could cause maximum muscle contraction. As shown in the experimental results, the rat hind leg started to slightly quiver when raising the current amplitude to 0.4 mA. Subsequently, the amplitude of both hind leg contraction and EMG signal gradually increased with the augmentation of current amplitude. While the current amplitude increased up to 1.6 mA and above, both the amplitude of hind leg contraction and the amplitude of EMG signal would not increase any more. Therefore, the threshold of current amplitude for full muscle recruitment could be set to 1.6 mA.

As shown in the EMG recording results (Fig. 4d), compared with bare gold electrodes, the noise amplitude and signal-to-noise ratio (SNR) for PEDOT/PSS modified electrodes was obviously reduced and improved, respectively. Moreover, after injecting of intramuscular lidocaine through the fluidic channels of the tubular microelectrodes, the muscle contraction obviously weakened under the same current amplitude. Meanwhile, the amplitude of EMG signal also reduced obviously after lidocaine injection, and the M-waves recorded by bare gold electrodes were mostly submerged in the noise and unable to be distinguished. As seen in the frequency spectra (Fig. 4e,f), the interference of signal recorded by PEDOT/PSS modified electrodes at 50 Hz was obviously lower than that of bare gold electrodes, which further indicated that the improved SNR of electrophysiological signal for PEDOT/PSS modified electrodes. Furthermore, comparing with the implantable microelectrodes based on rigid materials such as silicon or SU-8, the electrical stimulation and recording performance of the tubular microelectrode was barely changed during the electrophysiological experiments. At meantime, the muscle tissue was able to contract normally under repeated electrical stimulation by the tubular microelectrodes without bleeding. According to the *in vivo* experiment results, during the whole experiment process of the electrical stimulation induced repeated muscle contraction (lasted for several hours), the relative displacement of the implanted tubular microelectrodes was barely observed. Because of the good combination between the implanted microelectrodes and the muscle tissue, scarcely any obvious abnormity of muscle contraction was observed during the *in vivo* experiment. All these results demonstrated that our implantable tubular microelectrodes were suitable for dynamic electrophysiological recording and fluidic drug delivery *in vivo*, and the electrophysiological signal recording performance could be further enhanced by the PEDOT/PSS modification.

## Conclusion

In conclusion, we developed a flexible tubular microelectrode made of biocompatible polymer materials, which was suitable for dynamic tissue implantation. The tubular microelectrode provided electrical path for electrical stimulation and electrophysiological signal recording, as well as the chemical regulation channel for fluidic drug delivery. The 3D distribution of the electrode sites facilitated the improvement of spatial selectivity, and the way of the electrode sites distribution could be arbitrarily changed by the wrapping process and the pattern of the thin film electrode. Furthermore, the characteristics of the tubular microelectrode including electrochemical performance, mechanical property, flow resistance and biocompatibility were tested and verified to meet the requirements of implantable microelectrodes. Finally, the experimental results *in vivo* indicated that our implantable tubular microelectrodes were suitable for dynamic electrophysiological recording and fluidic drug delivery, and the electrode performance was further enhanced by the PEDOT/PSS modification. Comparing with the previous studies^23^,^24^,^25^, we further explored the enhanced electrochemical performance by conducting polymer coating, the mechanical stability during the repeated bending and *in vivo* experiments in dynamic muscle tissue to verify the feasibility of the tubular microelectrode for practical application. The tubular microelectrode has built multi-functional connections for tissue-machine interface, which can be minimized in dimension and integrated with more functions to provide precise and complex medical solutions in future.

## Methods

### Tubular microelectrode fabrication process

Some of the detailed fabrication processes for parylene thin film microelectrode have been described in our previous work^28^. As shown in Fig. S1, firstly, a layer of parylene C with thickness of 5 μm was chemical vapor deposited (CVD) on the silicon substrate by parylene deposition system (PDS 2010, SCS, USA). Then, a layer of photo resist was spin coated on the surface of parylene C, and the electrical leads pattern was formed by lithography. A layer of Cr/Au with thickness of 300 nm was sputtered on the patterned parylene C. Then, the extra metal and photoresist were removed by lift-off process. A second layer of parylene C with thickness of 5 μm was deposited to cover the metal leads. A second layer of photoresist was subsequently spin-coated and patterned to form the electrode sites, bonding pads and electrode boundary, respectively, which were then exposed by reactive ion etching. After removing from the substrate carefully, the parylene thin film microelectrode was helically wrapped on the surface of polyimide capillary (Bomi Co. Ltd, China) and adhered by tissue glue (3M, USA).

### Finite element analysis on the stimulation current

The Electric current model of AC/DC module in COMSOL software was chosen to simulate the stimulation current, as it solved ionic current distribution problems in electrolytes. The current in the domain was controlled by the continuity equation, which followed from Maxwell’s equations:


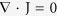


This equation used the following relations between the conductivity of the muscle tissue and the fields:


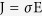


where *σ* was the conductivity of the muscle tissue. The conductivity of the muscle tissue was set as 5000 S/m (according to the COMSOL instruction). Ground potential boundary conditions were applied on the cylindrical surface (for the tubular microelectrode) or the square surface (for the flat microelectrode). The stimulating current density was set as 3000 A/m^2^ (applying 0.1 mA stimulating current on one electrode sites with diameter of 200 μm). All other boundaries were electrically insulated.

### Cell culture and immunofluorescent staining

In order to investigate the cell viability of the PEDOT/PSS covered parylene microelectrodes, the specific substrates for cells culture were developed. The substrates were fabricated by firstly covering the parylene film on the flat glass slides (4 mm × 4 mm). Then the substrates were sputtered with Au and deposited with PEDOT/PSS successively. Rat pheochromocytoma (PC-12) cells were employed in this study and purchased from Chinese Academy of Sciences. The PC-12 cells of 5 × 10^3^ were seeded onto samples of PEDOT/PSS covered parylene slices in 48-well flat-bottomed cell plates, incubated with DMEM (Gibco, USA) and supplied with 5% CO_2_ at 37 °C for 7 days. The neural growth factor (NGF, Sigma, USA) with concentration of 50 ng/ml was added on day 2 of the culture process. For observation of the cells morphology, cells were labeled after 7 days of cultivation. After the removal of the medium, the cells on samples were washed with PBS, and then fixed for 5 min in 3.5% formaldehyde in PBS. Then they were immersed in 0.1% Triton X-100 for 5 min, followed by washing in PBS for 3 times. After aforesaid processing, the cells on samples were stained with 3,30-dioctadecyloxacarbocyanine perchlorate (DiO, Fanbo, China) for 1 hour at room temperature without illumination. Subsequently, the nucleuses of these samples were quickly stained by 5 μg/ml DAPI (4,6-diamidino-2-phenylindole dihydrochloride, Sigma, USA) for 5 min at room temperature. Finally, the cells on samples were washed in vast PBS for removing residual stain, and viewed by a laser scanning confocal microscope (Leica TCS SP5, Leica, Germany).

### Electrochemical deposition

The conducting polymer electrolyte for electrode site coating was composed of 0.01 M 3,4-ethylenedioxythiophene (EDOT, Sigma-Aldrich, USA) and 0.05 M poly (sodium 4-styrenesulfonate) (PSS, Sigma-Aldrich, USA) in deionized water. Four electrode sites were connected to the working electrode of electrochemistry workstation (CHI660c, CH Instrument) separately, and a platinum foil was connected with the counter electrode and reference electrode together. The electrochemical deposition of PEDOT/PSS was performed in galvanostatic mode with deposition current density of 0.2 mA/cm^2^ for 600 seconds (thin film) and 1800 seconds (thick film).

### Electrochemical characterization

The electrochemical properties of bare gold micro wire electrodes and electrodes coated with PEDOT/PSS were characterized by electrochemical impedance spectrum (EIS) and cyclic voltammogram (CV). Both measurement processes were performed on electrochemistry workstation (CHI660c, CH Instrument) in phosphate buffered saline (PBS, pH 7.2–7.4) versus saturated calomel electrode (SCE, CH Instrument) as reference electrode and a platinum foil as counter electrode. EIS was measured at frequency ranging from 0.1 Hz to 100,000 Hz with input voltage amplitude of 0.01 V. CV was scanned over the potential range between −0.6 V and 0.8 V at the scanning rate of 50 mV/s. The EIS results were fitted to the equivalent circuit model for component calculation by using ZSimpWin software. The surface morphology of bare gold electrode and PEDOT/PSS coated electrode was observed by scanning electron microscope (SEM, ULTRA 55, Zeiss, Germany) at 5 kV.

### *In vivo* electrophysiological experiment

All surgical procedures were operated in accordance with protocols approved by the Shanghai Jiao Tong University Institution Animal Care and Use Committee (IACUC). All the animal experimental methods were carried out in accordance with the IACUC approved guidelines. All experimental protocols were approved by IACUC. Adult female Sprague-Dawley rats weighting about 360 g were chosen for intramuscular microelectrodes implantation, functional electrical stimulation and EMG recording experiments. The rats were anesthetized with chloral hydrate (4 mL/kg, 10% solution). The anesthetized condition was monitored by eye blink reflex during electrophysiological experiment process. A pair of wire electrodes (diameter of 100 μm) made of platinum and insulated by parylene C were used for sciatic nerve stimulation. The electrode site surface of the wire electrodes was electrodeposited with platinum black to improve electrochemical performance. The tubular microelectrodes were implanted into rat gastrocnemius (GA) and tibialis anterior (TA) with the help of syringe needles. The tubular microelectrodes with length of approximately 10 mm were implanted in the rat muscle tissue, which would directly interact with the muscle tissue. The electrophysiological experiments including electrical stimulation of sciatic nerve and EMG recording were operated with Multi-Channel Neurophysiology Workstation (RZ5D, Tucker-Davis Technologies, USA).

## Additional Information

**How to cite this article**: Tian, H.-C. *et al.* Enhanced Flexible Tubular Microelectrode with Conducting Polymer for Multi-Functional Implantable Tissue-Machine Interface. *Sci. Rep.* **6**, 26910; doi: 10.1038/srep26910 (2016).

## Supplementary Material

Supplementary Information

## Figures and Tables

**Table 1:** The numerical fitting results of equivalent circuit components of bare gold electrodes and PEDOT/PSS coated electrodes.

Items	*Z*(CPE)		*R*_*ct*_ (ohm)	*Z*_Warburg_		*C*_*d*_ (F)	χ^2^
	*Y*_0_ (S·sec^*n*^)	*n*		*W*_0_ (S·sec^0.5^)	*B*(sec^0.5^)		
Bare gold electrode	1.375 × 10^−8^	0.8129	1.059 × 10^16^	–	–	–	1.435 × 10^−2^
PEDOT/PSS thin film electrode	7.46 × 10^−5^	0.5062	587.6	7.104 × 10^−7^	1.256 × 10^−3^	6.086 × 10^−6^	2.871 × 10^−4^
PEDOT/PSS thick film electrode	2.045 × 10^−4^	0.1431	1.204 × 10^4^	1.066 × 10^−6^	2.373 × 10^−3^	1.814 × 10^−5^	1.886 × 10^−3^
