# Supplementary material for: Enhanced Flexible Tubular Microelectrode with Conducting Polymer for Multi-Functional Implantable Tissue-Machine Interface
Source: Sci Rep. 2016 May 27;6:26910. doi: 10.1038/srep26910 (PMC4882553; doi:10.1038/srep26910)
Supplement: Supplementary Information [file srep26910-s1.doc]

**Enhanced flexible tubular microelectrode with conducting polymer for multi-functional implantable tissue-machine interface**

Hong-Chang Tian ^1,2,3^, Jing-Quan Liu ^1,2,3^,*, Xiao-Yang Kang ^1,2,3^, Long-Jun Tang ^1,2,3^,

Ming-Hao Wang ^1,2,3^, Bo-Wen Ji ^1,2,3^, Bin Yang ^1,2,3^, Xiao-Lin Wang ^1,2,3^,

Xiang Chen ^1,2,3^ and Chun-Sheng Yang ^1,2,3^

^1^ National Key Laboratory of Science and Technology on Micro/Nano Fabrication Laboratory, Shanghai Jiao Tong University, Shanghai, PR China

^2^ Key Laboratory for Thin Film and Micro fabrication of Ministry of Education, Shanghai Jiao Tong University, Shanghai, PR China

^3^ Collaborative Innovation Center of IFSA, Department of Micro/Nano-electronics, Shanghai Jiao Tong University, Dongchuan Road 800, Shanghai, 200240, PR China

* Corresponding author. Tel.: +86 21 34207209; fax: +86 21 34207209.

*E-mail address*: jqliu@sjtu.edu.cn (J.Q. Liu).


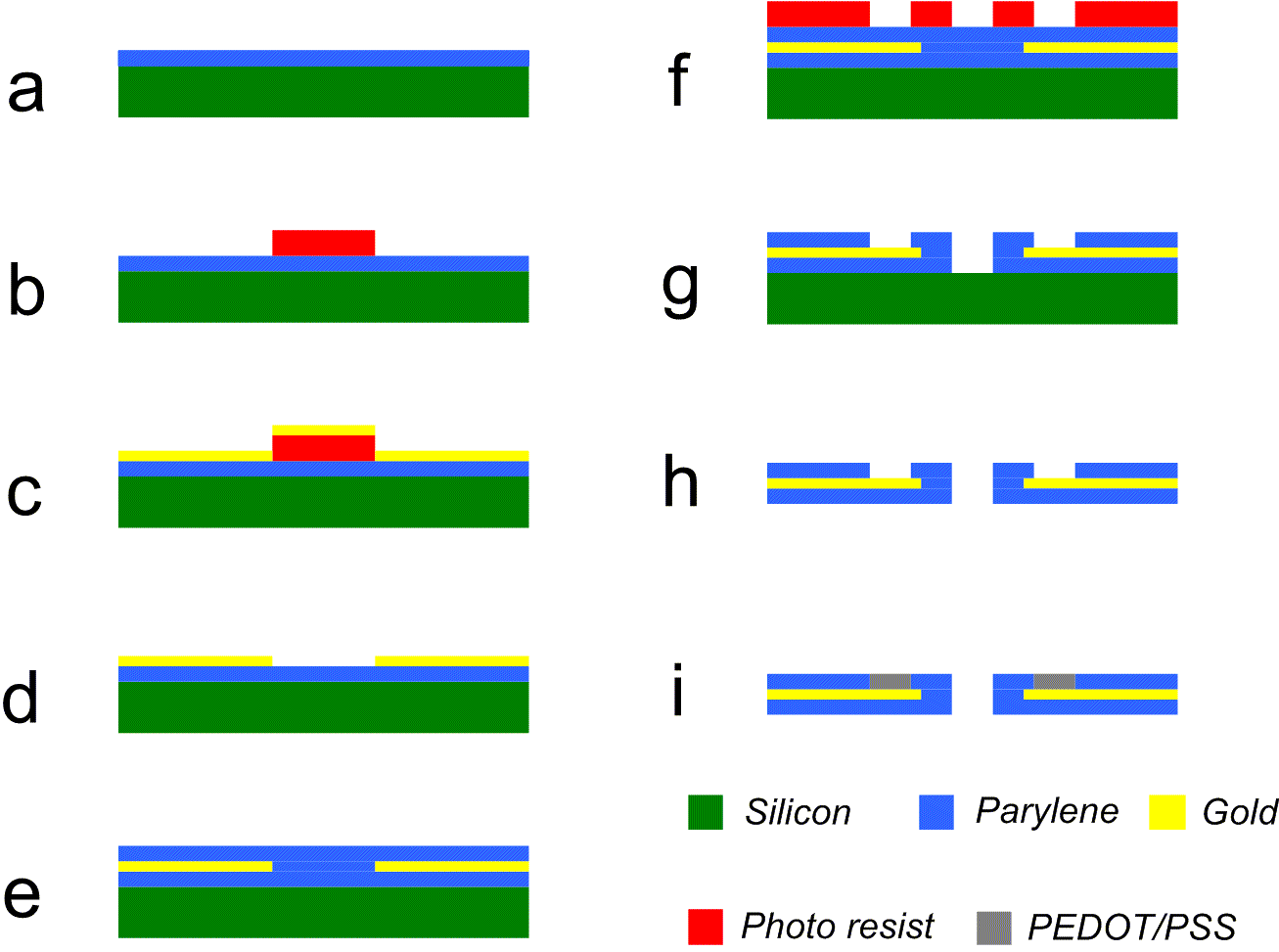


**Figure S1.** Fabrication process of the parylene based flexible electrode. a) Wafer cleaning and chemical vapor deposition (CVD) of the first layer of parylene; b) first spin coating and lithography of photo resist for patterning metallic path; c) sputtering Cr/Au; d) lift-off to form metallic conducting path; e) CVD of the second layer of parylene; f) second spin coating and lithography of photo resist for reactive ion etch (RIE); g) RIE to expose electrode sites and bonding pads; h) electrodes release from the substrate; i) electrochemically deposition of PEDOT/PSS for performance improvement


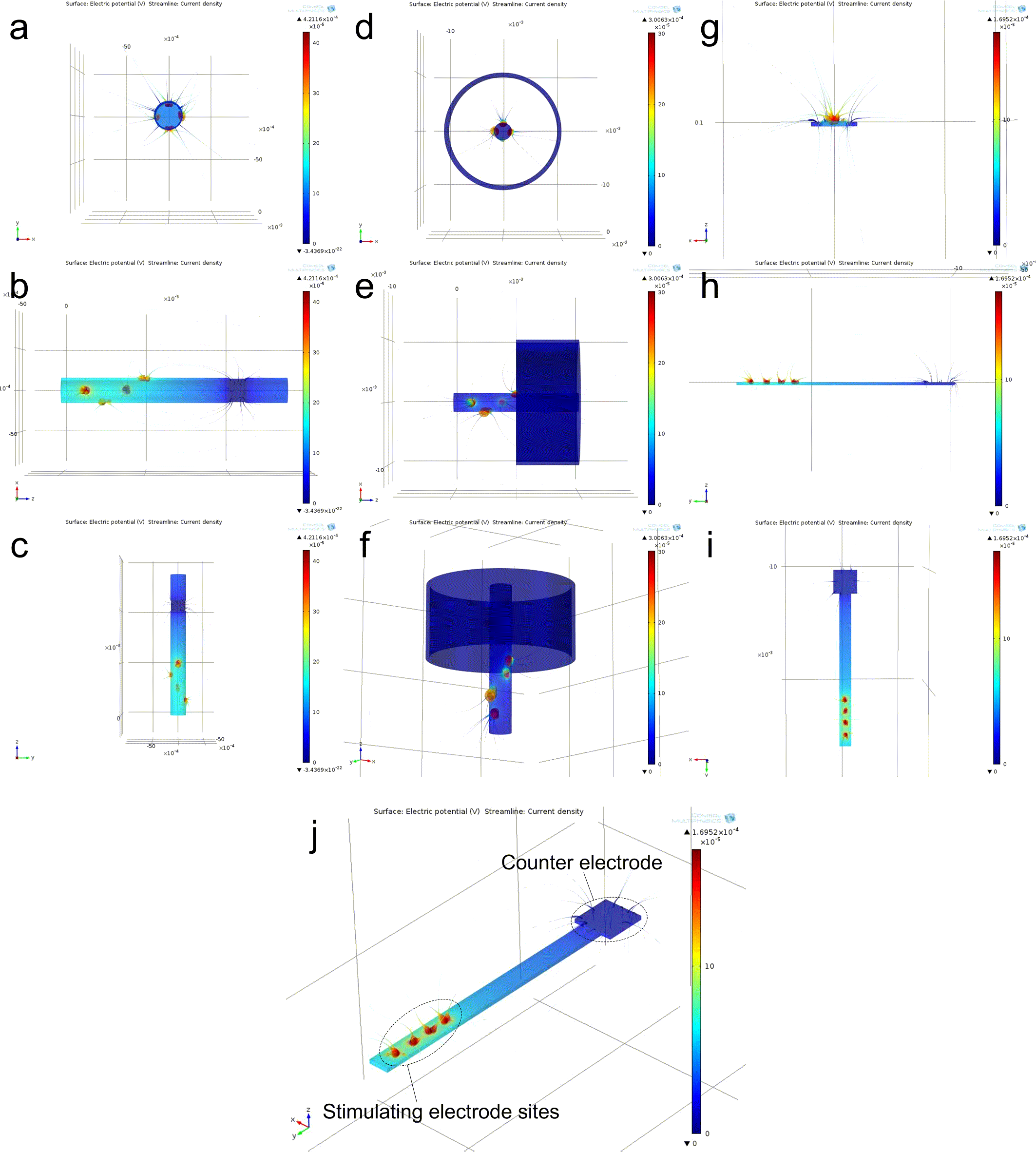


**Figure S2.** The finite element simulation result of stimulating current streamline distribution. a-c) the reference electrode was arranged on the tubular microelectrode. d-f) the reference electrode was arranged out of the muscle bundle. g-j) the flat microelectrode model.


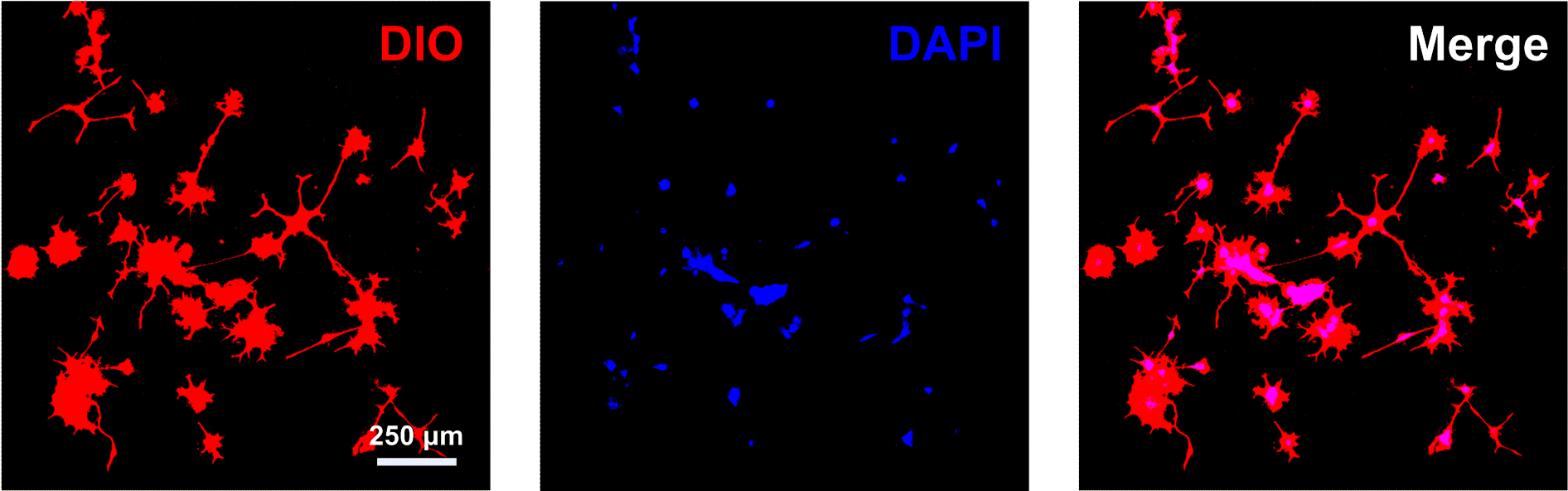


**Figure S3.** Morphology of PC-12 cells cultured on PEDOT/PSS coated parylene thin film electrode for 1 day observed by laser scanning confocal microscope (LSCM). The cell bodies and cell nucleuses were stained by 3,3′-dioctadecyloxacarbocyanine perchlorate (DIO) (red) and 4,6-diamidino-2-phenylindole dihydrochloride (DAPI) (blue), respectively.


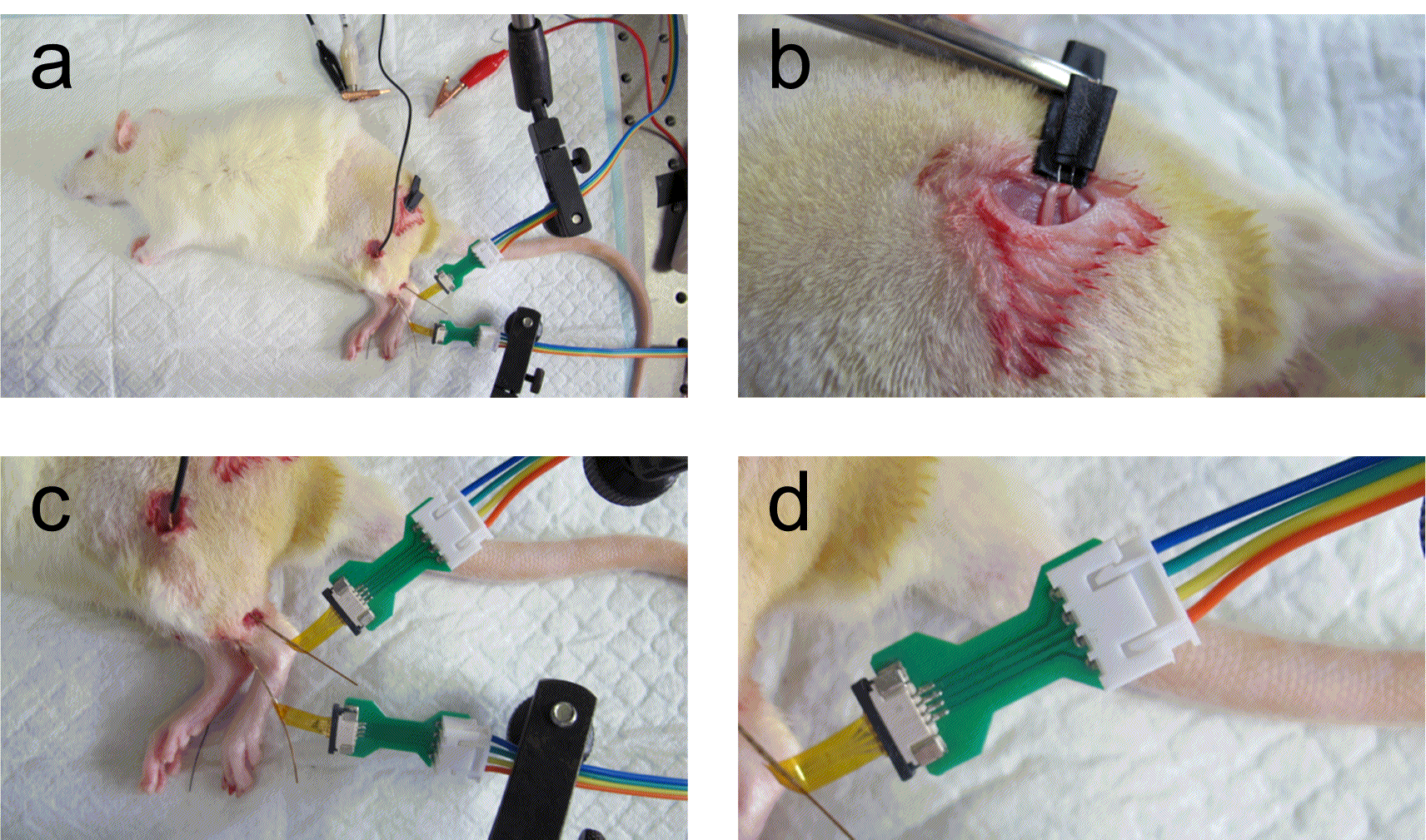


**Figure S4.** The actual configuration of the electrophysiological experiment. a) The overall configuration; b) the sciatic nerve stimulation part; c) the tubular microelectrodes implantation part; d) the electrode interface part.
